# Editorial: Insights of Gut Microbiota: Probiotics and Bioactive Compounds

**DOI:** 10.3389/fmicb.2021.780596

**Published:** 2021-11-01

**Authors:** Katia Sivieri, Sonia G. Sáyago-Ayerdi, Ana Griselda Binetti

**Affiliations:** ^1^School of Pharmaceutical Sciences, São Paulo State University (UNESP), Araraquara, Brazil; ^2^Tecnológico Nacional de México/Instituto Tecnológico de Tepic, Tepic, Mexico; ^3^Consejo Nacional de Investigaciones Científicas y Técnicas (CONICET) Instituto de Lactología Industrial (INLAIN) Santa Fe, Santa Fe, Argentina

**Keywords:** microbiome, functional foods, bioactive compound, prebiotic, probiotic

The human body harbors a multitude of microorganisms, including bacteria, fungi, archaea, and viruses, which exist in a symbiotic relationship with their host. The entirety of these commensals is referred to as the microbiota, and their collective genomic information as the human microbiome. The human microbiome has emerged as a crucial component in health and disease. It is becoming increasingly recognized that the microbiome can change our health status, or switch on a wide range of diseases including cancer, cardio-metabolic diseases, allergies, and obesity. In this way, probiotics and bioactive compounds may modulate the gut microbiota. The objective of this Research Topic is to show how probiotics and bioactive compounds can modulate the intestinal microbiota. Thus, research on gut microbiota and probiotics is moving from an isolated area to one with a range of possibilities. This Research Topic focuses on studies (including original research, perspectives, minireviews, commentaries, and opinion papers) that investigate and discuss: (1) The influence of probiotics on gut microbiota; (2) The influence of bioactive compounds on gut microbiota; (3) Methods, possibilities, and approaches to change and control gut microbiota, and (4) Experimental systems and approaches in gut microbiome research.

This collection includes 20 research articles spanning diverse publication formats, including 14 Original Research Articles, two Reviews, three Mini-reviews, and one Methods. Although different, the major articles have a similar objective of finding ways to modulate the intestinal microbiota.

The importance of probiotics in human health on gut microbiota modulation is indisputable. However, is there a consensus on what the quality criteria used for probiotics to be conveyed in food or in the form of supplements are? An interesting review from Binda et al., describes the minimum criteria that apply to a probiotic strain that will be used in foods and dietary supplements; similar criteria may be applicable to other uses of probiotics. These principles are based on the consensus statement from the International Scientific Association for Probiotics and Prebiotics (ISAPP) on the scope and appropriate use of the term probiotic. This document acts as a guide for both scientists and food producers serving as a tool to summarize the steps to consider when a potential probiotic strain is intended for use, ensuring the proper use of this term in scientific publications, on food product labels, and in regulatory documents. Furthermore, aiming at the health of gut microbiota, this special topic brings several important insights. Bengoa et al., showed that exopolysaccharides (EPS) produced by two *L. paracasei* strains isolated from kefir grains have the potential to improve the short chain fat acids' (SCFAs) production using an *in vitro* EPS fermentation by human fecal microbiota. In this way, Sabater et al., highlighted that EPS produced by *Bifidobacterium animalis* subsp. *lactis* can beneficially modify the gut microbiota with a potential immune modulation effect. On the other hand, Almada-Érix et al. showed that the consumption of yogurt containing *B. coagulans* GBI-30 6086 decreases triglycerides and glucose levels and positively impacts the gut bacterial ecology in healthy rats. Therefore, Li et al., showed on heat stroke in rats that a probiotic *Bacillus licheniformis* has a preventative effect on intestinal injury by sustaining intestinal barrier function and modulating gut microbiota. Interestingly, Diamond et al., demonstrated the beneficial potential of caffeine in microbiome and microbiome members during antibiotic exposure in animal model.

Another important topic we brought up was the importance of colonization of the intestinal microbiota in the first years of life. The delivery mode and the first 3 years of an infant's life are critical for the establishment of the intestinal microbiome. In this way, Lyu et al., evaluated how the delivery mode can affect the intestinal gut and development of intestinal epithelial cells. The authors conclude that vaginal delivery and cesarean section (C-section) can influence gut microbiota composition. In addition, they found that *B. bifidum* FL-228.1 exhibited favorable effects on the development of intestinal epithelial cell. In this sense, Cheng et al., highlighted that human milk oligosaccharides can promote the growth of *B. longum* subsp. *infantis*. Conversely, Yousuf et al., showed that routine in-hospital administration of probiotics to preterm infants resulted in the potential for colonization of the gut with probiotic post-discharge and effects on the gut microbiome, inducing the colonization by *Bifidobacterium*, thus ensuring that their intestinal microbiome resembles that of 10-day old full-term infants. Also, several large randomized controlled trials were revised by Murphy et al., showing that the relative risk for Necrotizing Enterocolitis can be reduced by the modulation of the gut microbiota with some (not all) probiotic formulations, highlighting the need to guarantee the purity and safety of commercially available probiotics, especially when they are intended to be implemented in a neonatal intensive care unit. On the other hand, the mini review realized by Ale and Binetti showed the clinical benefits of probiotic, prebiotic, and symbiotic consumption in the elderly, providing a better quality of life.

The action of probiotics and bioactive compounds on the microbiota of animals has also been investigated. Zhang et al., showed that ε-polylysine may influence the utilization of feed nutrients by Ningxiang pigs, including proteins, lipids, metabolizable energy, and fiber, by regulating the gut microbiota. In the same way, the paper published by Wang et al., deals with the effects of *Clostridium butyricum*, sodium butyrate, and butyric acid glycerides on the reproductive performance, egg quality, intestinal health, and offspring performance of yellow-feathered breeder hens; this document has already reached a milestone number of readers. The paper from Almada-Érix et al., has also been highly viewed by readers; it deals with orange juice and yogurt carrying probiotic *Bacillus coagulans* GBI-30 6086: Impact of intake on wistar male rats' health parameters and gut bacterial diversity. Something similar was discussed in the review proposed by González-Morelo et al. about molecular insights into O-linked glycan utilization by gut microbes, where the authors focus on the distinct molecular mechanism of consumption of these compounds from mucin and casein glycomacropeptide (GMP), highlighting the potential of these structures as emerging prebiotics.

Finally, this Research Topic shows significant advances made in the modulation of the gut microbiota in humans and animals, showing that the gut microbiota plays a fundamental role in health. On the other hand, the discoveries of microorganisms and key metabolites that make up the gut microbiota seem to us a future perspective for the development of effective clinical therapies. In this way, the results presented here may pave the way for creating effective clinical strategies using probiotics and bioactive compounds.

## Author Contributions

KS: writing, review, and edit. SS-A: writing. AB: writing and conceptualization. All authors contributed to the article and approved the submitted version.

## Conflict of Interest

The authors declare that the research was conducted in the absence of any commercial or financial relationships that could be construed as a potential conflict of interest.

## Publisher's Note

All claims expressed in this article are solely those of the authors and do not necessarily represent those of their affiliated organizations, or those of the publisher, the editors and the reviewers. Any product that may be evaluated in this article, or claim that may be made by its manufacturer, is not guaranteed or endorsed by the publisher.

